# Novel Toilet Paper–Based Point-Of-Care Test for the Rapid Detection of Fecal Occult Blood: Instrument Validation Study

**DOI:** 10.2196/20261

**Published:** 2020-08-07

**Authors:** Hsin-Yao Wang, Ting-Wei Lin, Sherry Yueh-Hsia Chiu, Wan-Ying Lin, Song-Bin Huang, Jason Chia-Hsun Hsieh, Hsieh Cheng Chen, Jang-Jih Lu, Min-Hsien Wu

**Affiliations:** 1 Department of Laboratory Medicine Chang Gung Memorial Hospital at Linkou Taoyuan City Taiwan; 2 PhD Program in Biomedical Engineering Chang Gung University Taoyuan City Taiwan; 3 Department of Health Care Management College of Management Chang Gung University Taoyuan City Taiwan; 4 Division of Hepatogastroenterology Department of Internal Medicine Kaohsiung Chang Gung Memorial Hospital Kaohsiung City Taiwan; 5 Syu Kang Sport Clinic Taipei City Taiwan; 6 Sigknow Biomedical Co Ltd Taipei City Taiwan; 7 Division of Oncology Department of Internal Medicine Chang Gung Memorial Hospital at Linkou Taoyuan City Taiwan; 8 Division of Oncology Department of Internal Medicine New Taipei Municipal TuCheng Hospital New Taipei City Taiwan; 9 School of Medicine Chang Gung University Taoyuan City Taiwan; 10 Department of Medical Biotechnology and Laboratory Science Chang Gung University Taoyuan City Taiwan; 11 Graduate Institute of Biomedical Engineering Chang Gung University Taoyuan City Taiwan; 12 Department of Chemical Engineering Ming Chi University of Technology New Taipei City Taiwan

**Keywords:** fecal occult blood test, point-of-care diagnostics, paper-based analytical devices, diagnostic, testing, detection, validation, cancer, public health

## Abstract

**Background:**

Colorectal cancer screening by fecal occult blood testing has been an important public health test and shown to reduce colorectal cancer–related mortality. However, the low participation rate in colorectal cancer screening by the general public remains a problematic public health issue. This fact could be attributed to the complex and unpleasant operation of the screening tool.

**Objective:**

This study aimed to validate a novel toilet paper–based point-of-care test (ie, JustWipe) as a public health instrument to detect fecal occult blood and provide detailed results from the evaluation of the analytic characteristics in the clinical validation.

**Methods:**

The mechanism of fecal specimen collection by the toilet-paper device was verified with repeatability and reproducibility tests. We also evaluated the analytical characteristics of the test reagents. For clinical validation, we conducted comparisons between JustWipe and other fecal occult blood tests. The first comparison was between JustWipe and typical fecal occult blood testing in a central laboratory setting with 70 fecal specimens from the hospital. For the second comparison, a total of 58 volunteers were recruited, and JustWipe was compared with the commercially available Hemoccult SENSA in a point-of-care setting.

**Results:**

Adequate amounts of fecal specimens were collected using the toilet-paper device with small day-to-day and person-to-person variations. The limit of detection of the test reagent was evaluated to be 3.75 µg of hemoglobin per milliliter of reagent. Moreover, the test reagent also showed high repeatability (100%) on different days and high reproducibility (>96%) among different users. The overall agreement between JustWipe and a typical fecal occult blood test in a central laboratory setting was 82.9%. In the setting of point-of-care tests, the overall agreement between JustWipe and Hemoccult SENSA was 89.7%. Moreover, the usability questionnaire showed that the novel test tool had high scores in operation friendliness (87.3/100), ease of reading results (97.4/100), and information usefulness (96.1/100).

**Conclusions:**

We developed and validated a toilet paper–based fecal occult blood test for use as a point-of-care test for the rapid (in 60 seconds) and easy testing of fecal occult blood. These favorable characteristics render it a promising tool for colorectal cancer screening as a public health instrument.

## Introduction

Colorectal cancer ranks globally as the third-leading and second-leading cancer type in terms of incidence and mortality rates, respectively. The incidence rate of colorectal cancer continues to rise because of aging populations and lifestyle changes. The continuous increase in its incidence rate has brought considerable health burdens worldwide [1-3]. Clinically, early screening of colorectal cancer in average-risk persons has been shown to reduce both mortality and incidence [4-7]. A variety of screening methods, including fecal occult blood testing, fecal immunochemical-based testing, sigmoidoscopy, digital rectal examination, colonoscopy, and computed tomographic (CT) colonography, have been developed for colorectal cancer screening [8]. However, the overall screening rate is still low [2], especially in areas with limited resources [9,10] or in socioeconomic minority groups [11-13]. In the United States, for example, the overall colorectal cancer screening rate was approximately 67.3% in 2016 [14]. In Taiwan, the screening rate was approximately 52.3% to 56.6% [15]. The low rate of colorectal cancer screening can be mainly attributed to the low availability, poor usability, or high cost of screening tools [9]. It is generally believed that the keys to successful implementation of colorectal cancer screening are cost-effectiveness, patient preference, and related professional medical resources [4,16,17]. Furthermore, it is important to provide flexibility and different choices for patients to conduct the examination themselves [18,19].

Among the screening methods described earlier, flexible sigmoidoscopy and colonoscopy are the gold standard primary screening methods [9,20,21] and can visually inspect the internal lining of the intestine [22]. By using endoscopy-based techniques, suspicious lesions or tumors can be detected, removed, and confirmed by pathology examination. However, scopy-based techniques require highly trained medical staff and specialized instruments. The operation of scopy-based instruments and the interpretation of the results are also highly operator dependent [23]. Moreover, the risks of scopy-based techniques include lower gastrointestinal bleeding, perforation, myocardial infarction, and ischemic stroke (approximately 5.3 per 10,000 persons; without biopsy or intervention, approximately 4.3 per 10,000 persons) [24,25]. CT colonography is a noninvasive exam for colorectal cancer screening with high sensitivity; it is comparable to colonoscopy [26,27]. Nevertheless, its widespread application is also limited by the availability of CT instruments and related facilities. High radiation exposure is another unfavorable feature of CT colonography [9,28]. Fecal occult blood tests and fecal immunochemical-based tests are both stool-based, noninvasive tests for colorectal cancer screening. Fecal occult blood testing targets heme, while fecal immunochemical-based testing detects hemoglobin in stool specimens. Studies have reported fecal immunochemical-based testing to have slightly higher performance than fecal occult blood testing in colorectal cancer screening [6,29]. However, the cost of fecal immunochemical-based testing is higher than that of fecal occult blood testing due to the expensive antihemoglobin antibody used in fecal immunochemical-based tests [30]. In terms of the successful implementation of colorectal cancer screening, fecal occult blood testing is the only screening method endorsed by the American Cancer Society to have the technical features of low cost and low medical profession requirements compared to other methods [6,31]. However, for current fecal occult blood testing, it is normally required that the test be performed by professional staff, restricting its application for point-of-care test or even home use. Moreover, current fecal occult blood tests generally require the user to collect fecal specimens using sticks to scoop stool after defecation. This unpleasant process could affect the widespread utilization of fecal occult blood tests for colorectal cancer screening [32-39].

To address this issue, we herein proposed a toilet paper–based fecal occult blood test (JustWipe) encompassing a toilet paper designed for fecal specimen collection and the reagents required for fecal occult blood test. For fecal specimen collection, we evaluated the working performance of the specially designed toilet paper. Moreover, we also developed and verified the analytical reagents used in the toilet paper–based tool. Based on the toilet paper design and analytical reagents, comparisons between the toilet paper–based tool and commercially available Hemoccult SENSA, as well as routine hospital fecal occult blood tests, were conducted to validate the new tool’s clinical utility and show that by using the novel toilet paper–based fecal occult blood test, we could easily collect fecal specimens in a regular buttocks-wiping move after defecation and detect fecal occult blood rapidly and accurately.

## Methods

### Design of the Toilet Paper-Based Fecal Specimen Collection Device

**Figure 1:**
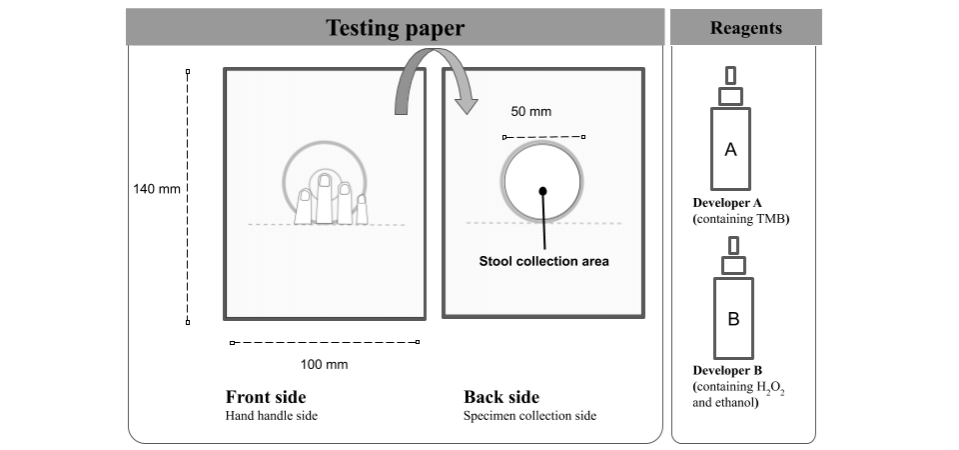
Design of the toilet paper-based fecal occult blood point-of-care test (JustWipe). TMB: 3,3’,5,5’-tetramethylbenzidine; H_2_O_2_: hydrogen peroxide.

**Figure 2:**
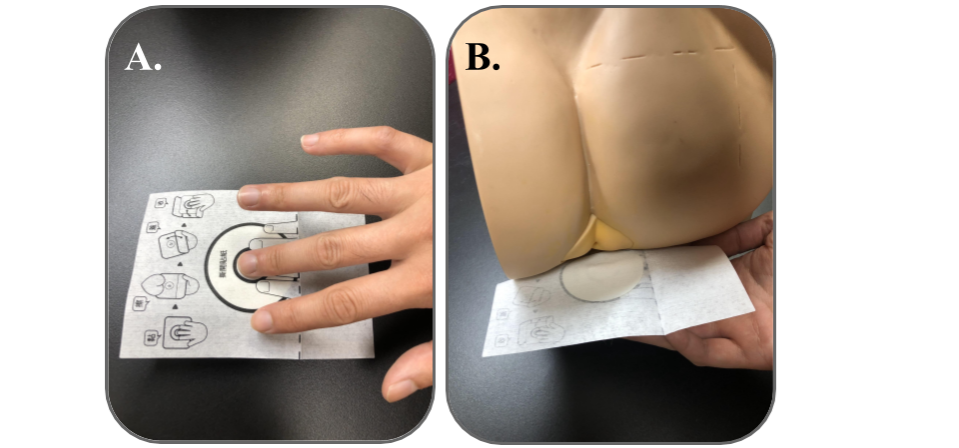
The appearance of the JustWipe: (A) The front side of the toilet testing paper with the hand handle side is clearly marked. (B) The backside of the toilet testing paper has a stool collection area with water repellent polyester cloth.

**Figure 3:**
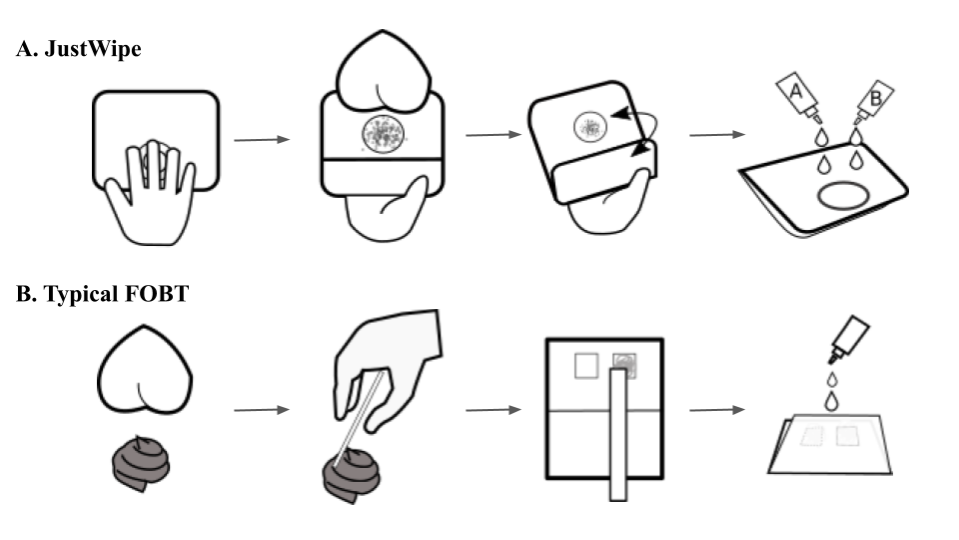
Comparison between JustWipe and typical fecal occult blood testing processes.

The toilet paper–based fecal occult blood test (JustWipe; Sigknow Biomedical Co Ltd) contained the testing paper for fecal specimen collection and the necessary reagents, as illustrated in Figure 1. Briefly, the specimen collection device was designed as a toilet paper–based tool. The user sticks his or her fingers onto the specific adhesive area on the front side of the paper and collects fecal specimens on the back side of the toilet paper during an ordinary buttocks-wiping move. The reagent part contains the two developers required for testing occult blood in the fecal specimen. Specifically, the toilet paper for fecal specimen collection (width: 100 mm, height: 140 mm) was composed of leaf bleached kraft pulp, which is commonly used for ordinary toilet paper. The front side (ie, hand handle side) was designed as a circular adhesive area (diameter: 50 mm) with a visual mark to facilitate correct handling of the tool (Figure 1 and Figure 2). The back side (ie, specimen collection side) was designed with a circular stool collection area (diameter: 50 mm) composed of a water repellent polyester cloth for fecal specimen collection (Figure 1 and Figure 2). Developer A contained 3,3’,5,5’-tetramethylbenzidine, and developer B contained hydrogen peroxide and ethanol. For JustWipe, the steps are to stick fingers onto the circular area of the front side, wipe buttocks, and collect fecal specimens on the circular area on the back side, fold the device, and apply reagents (developer A and developer B) to develop the test reaction, whereas for typical fecal occult blood tests, the steps are to defecate, scoop stool with stick, spread the collected stool on the test zone, and apply reagent to develop the test reaction. The entire process for JustWipe is schematically illustrated in comparison with that of the conventional fecal occult blood test counterpart in Figure 3. When occult blood exists in the specimen, a blue-green color develops. In contrast, a typical guaiac-based fecal occult blood test (eg, Hemoccult Sensa; Beckman Coulter) would require additional feces collection and spreading using sticks.

### Specimen Collection Performance of the Toilet Paper-Based Fecal Specimen Collection Device

To examine the variation in specimen collection using the toilet paper–based fecal specimen collection, 18 volunteers were recruited for the evaluation. For three consecutive days, each volunteer collected a specimen after defecation using the JustWipe method directly with a testing paper device. The weight of the testing paper was measured before and after collecting the specimen. The specimen weight from the testing paper was calculated by subtracting the testing paper weight before wiping from the testing paper weight after wiping.

### Limit of Detection of the Testing Reagents

We conducted a qualitative performance evaluation to determine the limit of detection of the reagents used in JustWipe according to Clinical and Laboratory Standards Institutes (CLSI) EP12-A2 User Protocol for Evaluation of Qualitative Test Performance [40]. Serial concentrations of hemoglobin solutions (0 µg/mL, 1.88 µg/mL, 2.26 µg/mL, and 3.75 µg/mL) consisting of human hemoglobin powder (Sigma-Aldrich) dissolved in double distilled water (ddH_2_O; Sigma-Aldric) were prepared. For each concentration, we applied 10 µL of hemoglobin solution to the circular collection area on the back side of the specimen collection device and then covered the circular collection area by folding the paper. Subsequently, we added 2 drops (80 µL) of reagent A and 2 drops (80 µL) of reagent B to develop the reaction for 60 seconds. Tests for each hemoglobin concentration were replicated 160 times (40 times by n=4 operators).

### Intraassay and Interassay Repeatability of the Testing Reagents

We evaluated both intraassay and interassay repeatability based on CLSI EP12-A2 [40]. We measured the performance of the testing reagents on different hemoglobin concentrations (0 µg/mL, 3.75 µg/mL, and 15 µg/mL) on three different days. Each hemoglobin concentration was tested 9 times on each independent day. We applied 10 µL of hemoglobin solution to the circular collection area on the back side of the specimen collection device and then covered the circular collection area by folding the paper. Subsequently, we added 2 drops (80 µL) of reagent A and 2 drops (80 µL) of reagent B to develop the reaction for 60 seconds. Tests of the same hemoglobin concentration performed on the same day were used to calculate the intraassay repeatability. In contrast, interassay repeatability was evaluated using the test results collected on different days.

### Reproducibility of the Testing Reagents in Untrained Users and Medical Staff

The reproducibility between untrained users (n=50) and trained medical staff (n=2) using the test reagents was evaluated based on CLSI EP5-A3 Evaluation of Precision Performance of Quantitative Measurement Methods guidance [41]. Two concentrations of hemoglobin solution (0 µg/mL and 3.75 µg/mL) were prepared and tested by the untrained users and trained medical staff. Each concentration of hemoglobin solution was tested twice.

### Performance Comparison Between JustWipe and the Typical Fecal Occult Blood Test in a Central Medical Laboratory

**Figure 4:**
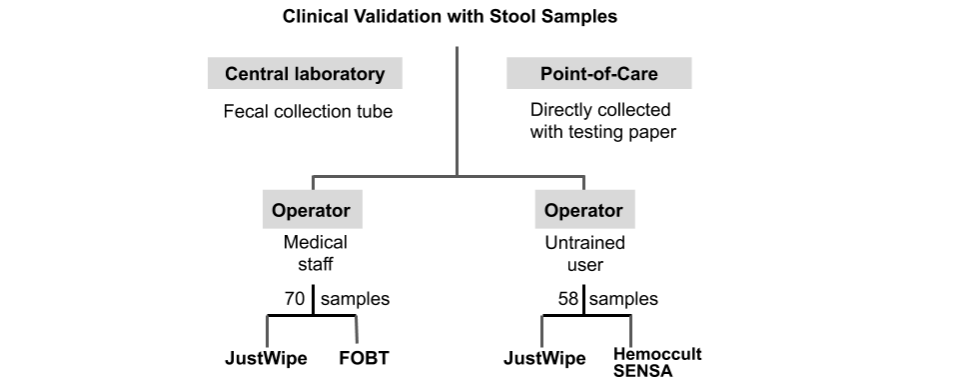
Study design flowchart. FOBT: fecal occult blood test.

We collected 70 convenience nonduplicate stool specimens from the central medical laboratory of Chang Gung Memorial Hospital Linkou branch between September 11, 2019 and March 24, 2020. The study was approved by the Institutional Review Board of Chang Gung Medical Foundation (No. 201901287B0). The stool specimens were collected in wards by nurses and placed into routine stool collection tubes [42]. The volume of the specimens was visually estimated to be greater than the size of a thumb. The specimens were deidentified before analytical measurements. An aliquot of stool was tested using a typical fecal occult blood test (O-tolidine test; Shin-Yung Medical Instruments Co Ltd). Another aliquot of stool was tested using JustWipe. The study design is illustrated in Figure 4.

### Performance Comparison Between JustWipe (as a Point-of-Care Test) and Hemoccult Sensa

We recruited 58 volunteers to use JustWipe as a point-of-care test in the period between March 1, 2019 and January 31, 2020. The volunteers were recruited from the Chang Gung Memorial Hospital and Chang Gung University. The study was approved by the Institutional Review Board of Chang Gung Medical Foundation (No. 201900133B0). The volunteers were asked to follow the instructions of JustWipe after defecation: the volunteer wiped his or her buttocks to collect stool specimens with the toilet paper–based fecal specimen collection device, followed by the steps of folding the device and applying the reagents. The volunteers interpreted the results themselves after 60 seconds of reaction. Subsequently, for testing with Hemoccult SENSA, the volunteers collected the rest of the specimens (a volume greater than that of a thumb) and sent the specimens to the medical staff within 20 hours which were kept at room temperature or stored between 2 ℃ to 8 ℃ and shipped to the laboratory next day. For Hemoccult SENSA (Beckman Coulter), the stool specimens received by the laboratory were then tested by medical staff according to the instructions of Hemoccult SENSA.

### Usability and User Preference Evaluation of JustWipe

The questionnaire was designed to assess the usability and user preference of the JustWipe. The questionnaire contained 12 questions about the user’s experience of the toilet paper–based fecal occult blood test (Table 1). Each question had 5 different response options (strongly agree, agree, neutral, disagree, and strongly disagree), which reflected the volunteers’ feedback on the usability of the test. The 58 recruited volunteers from the above study were requested to fill in the questionnaire by themselves after using JustWipe.

**Table 1:** Questionnaire used for usability evaluation. There were 12 questions on the questionnaire to assess the three aspects of the test, namely, operation friendliness, ease of reading the results, and information usefulness.

Group	Number	Items	Average agreement, %
Information usefulness	1	Did you know that the detection target is fecal occult blood?	100
	2	Did you know that the testing result is not an indicator of cancer?	98.6
	3	Did you know that the testing result is only for physical conditions?	100
	4	Do you know what to do after testing?	88.3
	5	If the test result is positive, would you go to the hospital for further examination?	93.8
Operational friendliness	6	Are the two bottle designs easy to identify?	89.0
	7	Do you understand all of the items in the device using the instruction manual?	86.2
	8	Is detailed company information provided in the instruction manual?	87.2
	9	Are all cautions clearly presented to the user?	87.6
	10	Are the operational procedures clearly presented to the user through icons and words?	86.9
Ease of result reading	11	Is your interpretation of the circle window the same as the medical staff’s result?	95.2
	12	Is your interpretation of the control area the same as the medical staff’s result?	99.7

### Statistical Analysis

We used a one-way analysis of variance (ANOVA) to test weight differences among specimens collected on different days. Kruskal-Wallis one-way ANOVA was used to test weight differences among specimens collected by different individuals. The statistical analysis of the two diagnostic test evaluation studies followed the statistical guidance on reporting results from studies evaluating diagnostic tests [43]. The 2×2 tables for each study were produced for comparisons between the index test method (ie, JustWipe) and the comparative methods. The estimation of the agreements included positive agreement, negative agreement, and overall agreement. Approximate 95% confidence limits for the true overall, positive, and negative agreement were calculated as the estimated value ± 2 standard error. The method for standard error calculation followed EP12-P from National Committee for Clinical Laboratory Standards [44].

## Results

### Variation in Specimen Collection is Acceptable Among Different Individuals and Different Days

**Figure 5:**
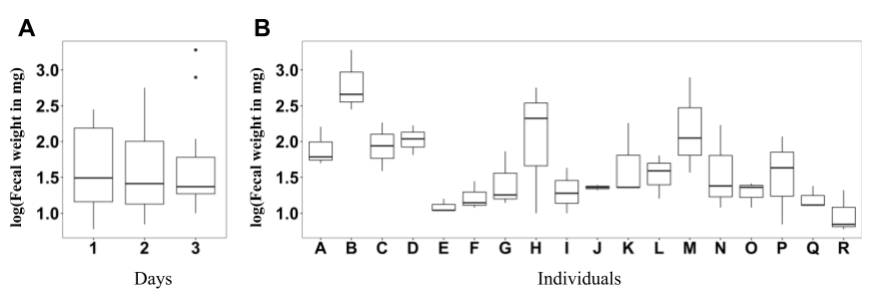
Weight distribution and variation of fecal specimens that are collected among (A) different days and (B) different users.

We evaluated the variation of specimen collection when using the toilet paper–based fecal specimen collection device (Figure 5). Both between-day variation and between-individual variation were evaluated in 18 individuals. In total, the median specimen weight was 25 mg, and the interquartile range was 89 mg. The minimum specimen weight was 6 mg, and the maximum weight was 1897 mg. Regarding the between-day variation, there was no difference in the weight of specimens collected on the 3 different days (*P*=.12). In contrast, a significant specimen weight difference was noted for different individuals. Specifically, 3 out of the 18 individuals collected a significantly greater amount (as a specimen) than the others did. When these 3 individuals were excluded, the statistical test showed no specimen weight differences among individuals. In summary, between-day variation was not significant. Between-individual variation was significant though, the outliers collected more specimen than the averages.

### Analytical Characteristics of the Test Reagents

**Figure 6:**
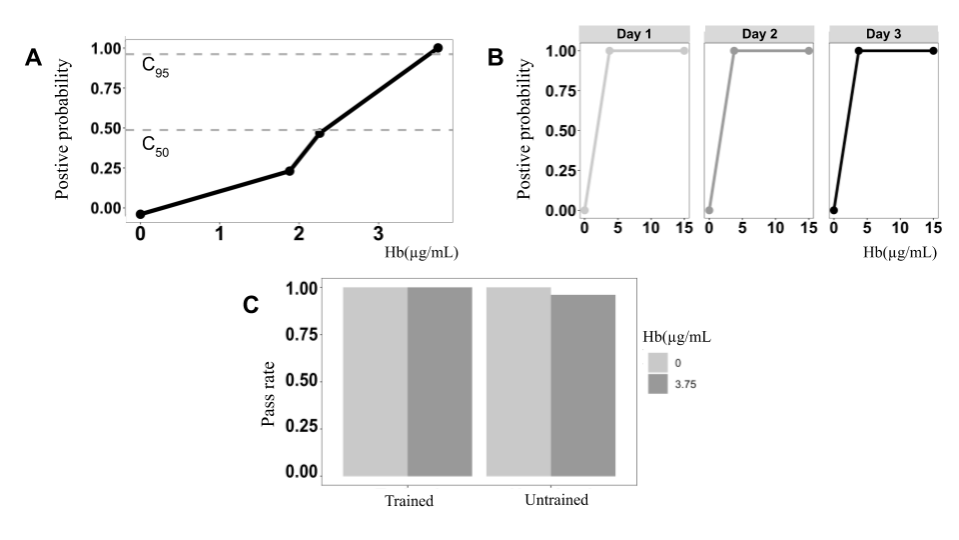
Analytical characteristics of the test reagents: (A) limit of detection, (B) positive probabilities for 5 and 15 µg/mL, and (C) reproducibility among trained (medical staff) or untrained users.

An imprecision curve of the analytical reagents used in JustWipe was used to illustrate the analytical characteristics (Figure 6). The probabilities of positive results for 2.26 µg/mL and 3.75 µg/mL were 48.8% and 98.8%, respectively. The concentration of 2.26 µg/mL could be defined as C50 (at which yielded 50% positive result and 50% negative result) for the test reagents [43]. The concentration of 3.75 µg/mL could be defined as the limit of detection (equal to C95) at which over 95% of the samples tested positive. The repeatability of testing different hemoglobin levels on different days is demonstrated in Figure 6. The positive probabilities for 5 µg/mL and 15 µg/mL were 100% for each over the three days; for 0 µg/mL, the positive probability was 0% over the three days. The repeatability for a longer period (20 days) is shown in Multimedia Appendix 1. Moreover, the reproducibility across untrained individuals and medical staff is illustrated in Figure 6. The trained users correctly operated all the samples whose concentrations were either 0 µg/mL or 3.75 µg/mL; the untrained users correctly operated the samples of 0 µg/mL, but the pass rate for 3.75 µg/mL was 96.0% (48/50).

### Clinical Validation of JustWipe in a Central Laboratory Setting

A comparison of the performance between JustWipe and a typical fecal occult blood test (O-tolidine–based test) was conducted using 70 clinical specimens collected in a tertiary referral hospital. The overall agreement was 82.9% (52/70); the positive agreement and negative agreement were 83.9% (26/31) and 82.1% (32/39), respectively (Table 2). The qualitative test results of both methods can be found in Multimedia Appendix 2.

**Table 2:** Performance comparison between JustWipe and a typical fecal occult blood test, and between JustWipe and Hemoccult SENSA.

Comparison		Hospital fecal occult blood test							Hemoccult SENSA				
			Positive		Negative		Total		Positive		Negative		Total
**JustWipe, n**													
	Positive		26		7		33		16		2		18
	Negative		5		32		37		4		36		40
	Total		31		39		70		20		38		58
**Agreement, %**													
	Estimate (95% CL^a^)		83.9 (70.7, 97.1)		82.1 (69.8, 94.3)		82.9 (73.9, 91.9)		80.0 (62.1, 80.0)		94.7 (87.5, 102)		89.7 (81.7, 97.7)

^a^95% confidence limits calculated as (estimate – 2 standard error, estimate + 2 standard error).

### Validation of JustWipe as a Point-Of-Care Test

The intended use of JustWipe is testing occult blood in the stool at home or at a point of care. Thus, we evaluated the performance of JustWipe when it was used as a point-of-care test according to the manufacturer’s instructions. The overall agreement was 89.7% (52/58); the positive agreement and negative agreement were 80.0% (16/20) and 94.7% (36/38), respectively (Table 2). The qualitative test results of both methods can be found in Multimedia Appendix 3.

### Usability and User Preference Evaluation of JustWipe

Of the 58 volunteers, there were 32 (55.2%) women and 26 (44.9%) men. The average age of the volunteers was 59.1 (SD 12.7) years. We asked the volunteers to complete the questionnaire (12 questions included) after using JustWipe as a point-of-care test. We summarized the results of the questionnaire into three categories: information usefulness (Q1, Q2, Q3, Q4, and Q5), operational friendliness (Q6, Q7, Q8, Q9, and Q10), and ease of reading results (Q11 and Q12). In terms of information usefulness, average agreement percentages for Q1, Q2, Q3, Q11, and Q12 were 100%, 98.6%, 100%, 88.3%, and 93.8%, respectively. In the category of operational friendliness, average agreement percentages for Q6, Q7, Q8, Q9, and Q10 were 89%, 86.2%, 87.2%, 87.6%, and 86.9%, respectively. For the category of ease of reading results, Q11 had an average of 95.2% and Q12 had an average of 99.7%.

## Discussion

### Principal Findings

In this study, we developed a toilet paper–based point-of-care test (JustWipe) for the rapid detection of fecal occult blood. An ordinary buttocks-wiping move on the toilet was adapted as the mechanism of stool specimen collection. Specimen collection by buttocks-wiping was evaluated to be useful and stable. In addition, a set of test reagents was developed and characterized. We compared the performance of JustWipe with that of a typical fecal occult blood test in a central laboratory setting. Moreover, we also demonstrated the performance of JustWipe as a point-of-care test. Based on the toilet paper–based device, stool specimens can be collected with a regular buttocks-wiping move. A rapid test result is available within 60 seconds using the test reagents. An ordinary user can operate the test easily, rapidly, and with reproducibility. The ease of operation and reliability render the novel fecal occult blood test a promising tool for colorectal cancer screening.

Troublesome operation of stool specimen collection is considered one of the obstacles affecting participation in colorectal cancer screening [13,32,34,36,37,39]. In a typical fecal occult blood test, fecal immunochemical-based test, or other stool-based occult blood tests, stool specimen collection is difficult. Users, typically with a nonmedical background, must sample stool by themselves. Several steps, including flushing the toilet bowl and floating tissue paper on the surface of the toilet bowl water, are required to be followed and performed correctly to ensure the quality of the specimen [45]. The users must also collect stool samples before it comes into contact with the toilet bowl water [45]. The demanding requirements not only reduce willingness to use the device but also result in some analytical errors when some of the steps are not performed correctly. In contrast, the fecal specimen collection using JustWipe requires a regular buttocks-wiping move only. The number of steps in fecal specimen collection is reduced so that the errors occurring in specimen collection can be largely mitigated. Moreover, the toilet paper–based stool specimen collection was shown to be stable between days and among different users (Figure 5). The mean and median weights of the stool specimens were 120.00 mg and 25.50 mg, respectively. The amount of specimen collected was higher than that collected via typical methods. The relationship between the amount of stool specimen to test sensitivity is not clear. However, a larger amount collected as a specimen is thought to be an advantageous feature for a test [46].

The analytical evaluation of the test reagents used in JustWipe demonstrated several favorable characteristics, including high sensitivity, high repeatability, and high reproducibility. Low-concentration hemoglobin could be detected with minimal day-to-day variation (high repeatability) and person-to-person variation (high reproducibility). Regarding the analytical sensitivity of the test reagents, the test reagents were found to have adequate analytical sensitivity to detect occult fecal blood in colorectal cancer patients. In a population study including 5.8 million individuals, a hemoglobin concentration of approximately 20 µg/mL could detect colorectal cancer with a detection rate of 5.2% in men and 2.2% in females. The hemoglobin concentration for the colorectal cancer patients identified in that study was between 172.8 and 231 µg hemoglobin/g feces [47]. In another study, a cut-off of 80-90 µg hemoglobin/g feces was sufficient for clinical application [48-50]. Based on the reported values (ie, 80-90 µg hemoglobin/g feces), a cut-off of 114.3-128.6 µg hemoglobin/mL is clinically useful when the water content in stool is 70% [51]. In contrast, the hemoglobin concentration in the feces of healthy individuals without colorectal cancer is 0.519 µg hemoglobin/mL (90% CI 0.468-0.575) in men and 0.283 µg hemoglobin/mL (90% CI 0.257-0.316) in women [50,52,53]. In brief, the limit of detection (3.75 µg hemoglobin/mL) of the test reagents of toilet paper–based tool was sensitive and stable enough for detecting fecal occult blood in colorectal cancer patients (Figure 6).

We validated JustWipe in the settings of a central laboratory and point of care. In both settings, JustWipe showed high agreement with typical test methods. In the setting of the central laboratory, both JustWipe and the comparative test (O-tolidine–based fecal occult blood test) were performed by medical staff. The positive agreement (83.9%) and negative agreement (82.1%) were quite balanced (Table 2). In contrast, the positive agreement (80.0%) was significantly lower than the negative agreement (94.7%) when we validated JustWipe used as a point-of-care test (Table 2). To validate JustWipe as a point-of-care test, the comparative method (Hemoccult SENSA) was operated by medical staff, while JustWipe was operated by nonmedical individuals. Regarding the association between the usability and the discordance between positive and negative agreement of the test results, we used Fisher exact test for the analysis. The results in Multimedia Appendix 4 showed that the usability indicators (Table 1) were not associated with the discordance of the test results. The specific usability indicators should have been associated with the discordant results. However, based on our data, the significant association was not detected in the study. The nonsignificant association could be attributed to the relatively small sample size used in the proof-of-concept validation. Yet, the association is worthy of further investigation as a key for improving the proposed device. Furthermore, the possible cause for the suboptimal positive agreement could be attributed to false negative interpretation of the weak positive reaction. Users who are not trained medical professionals may tend to ignore the weak signal on the toilet paper. To address the limitation of interpretation, especially in the weak positive case, we plan to develop an artificial intelligence–aided interpretation tool. By using the artificial intelligence–aided interpretation tool, images of the test result can be interpreted with a standardized approach. The interpretative error resulting from insufficient interpretation experience would be largely mitigated. We illustrate the approach in Multimedia Appendix 5.

The major aim of designing JustWipe was to improve the usability of fecal occult blood testing. We assessed the usability of JustWipe using a questionnaire (Table 1). The categories of operational friendliness and ease of reading results are important indicators for a nonmedical professional using a point-of-care test [37,39]. Regarding operational friendliness, the agreement of all the questions (Q6, Q7, Q8, Q9, and Q10) was greater than 80% (range 81.9%-84.5%). For ease of reading results (Q11 and Q12), the agreement was greater than 94%. In brief, the high agreement in operational friendliness and ease of reading results indicated that users without professional medical training can easily operate the tool and interpret it following the instructions.

### Limitations

Although the volunteers from our trial in the point-of-care test setting would be representative of the target population with respect to the range of age, sampling bias could still exist in the validation setting for the point-of-care test trial. All volunteers were recruited from the urban region in northern Taiwan. The average age of the recruited volunteers in the point-of-care test was 59.1 (SD 12.7) years. The range of age was approximately the age of the target population for most cancer screening programs. In the majority of Europe, the colorectal cancer prevention program recommends screening for people above 50 years old [54]. The US Preventive Services Task Force also recommend screening the population above 50 years old [6]. The colorectal cancer screening program in Taiwan also recommends screening for the population above 50 years old [55]. In this study, the volunteers were recruited from one tertiary medical center (ie Chang Gung Memorial Hospital, Linkou branch) and one university (ie, Chang Gung University). The volunteers may have had chronic diseases and subclinical conditions. The preliminary validation results reported in the study could not be easily applied to other populations. The performance using the proposed device in general population needs further investigation in a larger cohort. The demographic characteristics of the 58 recruited volunteers are listed in Multimedia Appendix 6.

### Conclusions

We developed and validated a toilet paper–based point-of-care test for detecting fecal occult blood. The result showed that the toilet paper–based collection of fecal specimens was stable. The test reagents of the point-of-care test also showed high repeatability and reproducibility. The novel toilet paper–based point-of-care test revealed high agreement with the comparative methods in both central laboratory and point-of-care test settings. The usability evaluation of the point-of-care test showed high operation friendliness and high ease of reading results. The favorable characteristics render the proposed novel point-of-care test a promising colorectal cancer screening tool.

## Appendix

Multimedia Appendix 1 Repeatability for a longer period (20 days) for the analytical characteristics of the test reagents.

Multimedia Appendix 2 Qualitative test results for both methods in the central laboratory.

Multimedia Appendix 3 Qualitative test results for both methods in the point-of-care test setting.

Multimedia Appendix 4 Association between individual usability indicators and result discordancy. The table showed the contingency table for each question on recruited volunteers with discordance and concordance result.

Multimedia Appendix 5 Using the mobile device for interpretation of test results. The workflow demonstrates how to use a mobile phone to assist interpretation with artificial intelligence model prediction.

Multimedia Appendix 6 This table contains the demographic characteristics of age, gender, education, and occupation of the recruited volunteers.

## Abbreviations

ANOVA: analysis of variance
CLSI: Clinical and Laboratory Standards Institutes
CT: computed tomographic
