# Supplementary material for: Novel Toilet Paper–Based Point-Of-Care Test for the Rapid Detection of Fecal Occult Blood: Instrument Validation Study
Source: J Med Internet Res. 2020 Aug 7;22(8):e20261. doi: 10.2196/20261 (PMC7472847; doi:10.2196/20261)
Supplement: Multimedia Appendix 1 [file jmir_v22i8e20261_app1.docx]

# **Supplementary Appendix**

### **Table S1. The repeatability and reproducibility test for 20 continuous days from two operators**

S1-a. Testing result of operator 1 (Lot. A18 L01)

|  | Day 1 | | Day 2 | | Day 3 | | Day 4 | |
| --- | --- | --- | --- | --- | --- | --- | --- | --- |
|  | 3.75 µg/ml | 0 µg/ml | 3.75 µg/ml | 0 µg/ml | 3.75 µg/ml | 0 µg/ml | 3.75 µg/ml | 0 µg/ml |
| Run 1 | Pass | Pass | Pass | Pass | Pass | Pass | Pass | Pass |
|  | Pass | Pass | Pass | Pass | Pass | Pass | Pass | Pass |
| Run 2 | Pass | Pass | Pass | Pass | Pass | Pass | Pass | Pass |
|  | Pass | Pass | Pass | Pass | Pass | Pass | Pass | Pass |
|  | Day 5 | | Day 6 | | Day 7 | | Day 8 | |
|  | 3.75 µg/ml | 0 µg/ml | 3.75 µg/ml | 0 µg/ml | 3.75 µg/ml | 0 µg/ml | 3.75 µg/ml | 0 µg/ml |
| Run 1 | Pass | Pass | Pass | Pass | Pass | Pass | Pass | Pass |
|  | Pass | Pass | Pass | Pass | Pass | Pass | Pass | Pass |
| Run 2 | Pass | Pass | Pass | Pass | Pass | Pass | Pass | Pass |
|  | Pass | Pass | Pass | Pass | Pass | Pass | Pass | Pass |
|  | Day 9 | | Day 10 | | Day 11 | | Day 12 | |
|  | 3.75 µg/ml | 0 µg/ml | 3.75 µg/ml | 0 µg/ml | 3.75 µg/ml | 0 µg/ml | 3.75 µg/ml | 0 µg/ml |
| Run 1 | Pass | Pass | Pass | Pass | Pass | Pass | Pass | Pass |
|  | Pass | Pass | Pass | Pass | Pass | Pass | Pass | Pass |
| Run 2 | Pass | Pass | Pass | Pass | Pass | Pass | Pass | Pass |
|  | Pass | Pass | Pass | Pass | Pass | Pass | Pass | Pass |
|  | Day 13 | | Day 14 | | Day 15 | | Day 16 | |
|  | 3.75 µg/ml | 0 µg/ml | 3.75 µg/ml | 0 µg/ml | 3.75 µg/ml | 0 µg/ml | 3.75 µg/ml | 0 µg/ml |
| Run 1 | Pass | Pass | Pass | Pass | Pass | Pass | Pass | Pass |
|  | Pass | Pass | Pass | Pass | Pass | Pass | Pass | Pass |
| Run 2 | Pass | Pass | Pass | Pass | Pass | Pass | Pass | Pass |
|  | Pass | Pass | Pass | Pass | Pass | Pass | Pass | Pass |
|  | Day 17 | | Day 18 | | Day 19 | | Day 20 | |
|  | 3.75 µg/ml | 0 µg/ml | 3.75 µg/ml | 0 µg/ml | 3.75 µg/ml | 0 µg/ml | 3.75 µg/ml | 0 µg/ml |
| Run 1 | Pass | Pass | Pass | Pass | Pass | Pass | Pass | Pass |
|  | Pass | Pass | Pass | Pass | Pass | Pass | Pass | Pass |
| Run 2 | Pass | Pass | Pass | Pass | Pass | Pass | Pass | Pass |
|  | Pass | Pass | Pass | Pass | Pass | Pass | Pass | Pass |

S1-b. Testing result of operator 2 (Lot. A18 L01)

|  | Day 1 | | Day 2 | | Day 3 | | Day 4 | |
| --- | --- | --- | --- | --- | --- | --- | --- | --- |
|  | 3.75 µg/ml | 0 µg/ml | 3.75 µg/ml | 0 µg/ml | 3.75 µg/ml | 0 µg/ml | 3.75 µg/ml | 0 µg/ml |
| Run 1 | Pass | Pass | Pass | Pass | Pass | Pass | Pass | Pass |
|  | Pass | Pass | Pass | Pass | Pass | Pass | Pass | Pass |
| Run 2 | Pass | Pass | Pass | Pass | Pass | Pass | Pass | Pass |
|  | Pass | Pass | Pass | Pass | Pass | Pass | Pass | Pass |
|  | Day 5 | | Day 6 | | Day 7 | | Day 8 | |
|  | 3.75 µg/ml | 0 µg/ml | 3.75 µg/ml | 0 µg/ml | 3.75 µg/ml | 0 µg/ml | 3.75 µg/ml | 0 µg/ml |
| Run 1 | Pass | Pass | Pass | Pass | Pass | Pass | Pass | Pass |
|  | Pass | Pass | Pass | Pass | Pass | Pass | Pass | Pass |
| Run 2 | Pass | Pass | Pass | Pass | Pass | Pass | Pass | Pass |
|  | Pass | Pass | Pass | Pass | Pass | Pass | Pass | Pass |
|  | Day 9 | | Day 10 | | Day 11 | | Day 12 | |
|  | 3.75 µg/ml | 0 µg/ml | 3.75 µg/ml | 0 µg/ml | 3.75 µg/ml | 0 µg/ml | 3.75 µg/ml | 0 µg/ml |
| Run 1 | Pass | Pass | Pass | Pass | Pass | Pass | Pass | Pass |
|  | Pass | Pass | Pass | Pass | Pass | Pass | Pass | Pass |
| Run 2 | Pass | Pass | Pass | Pass | Pass | Pass | Pass | Pass |
|  | Pass | Pass | Pass | Pass | Pass | Pass | Pass | Pass |
|  | Day 13 | | Day 14 | | Day 15 | | Day 16 | |
|  | 3.75 µg/ml | 0 µg/ml | 3.75 µg/ml | 0 µg/ml | 3.75 µg/ml | 0 µg/ml | 3.75 µg/ml | 0 µg/ml |
| Run 1 | Pass | Pass | Pass | Pass | Pass | Pass | Pass | Pass |
|  | Pass | Pass | Pass | Pass | Pass | Pass | Pass | Pass |
| Run 2 | Pass | Pass | Pass | Pass | Pass | Pass | Pass | Pass |
|  | Pass | Pass | Pass | Pass | Pass | Pass | Pass | Pass |
|  | Day 17 | | Day 18 | | Day 19 | | Day 20 | |
|  | 3.75 µg/ml | 0 µg/ml | 3.75 µg/ml | 0 µg/ml | 3.75 µg/ml | 0 µg/ml | 3.75 µg/ml | 0 µg/ml |
| Run 1 | Pass | Pass | Pass | Pass | Pass | Pass | Pass | Pass |
|  | Pass | Pass | Pass | Pass | Pass | Pass | Pass | Pass |
| Run 2 | Pass | Pass | Pass | Pass | Pass | Pass | Pass | Pass |
|  | Pass | Pass | Pass | Pass | Pass | Pass | Pass | Pass |
