# Supplementary material for: Novel Toilet Paper–Based Point-Of-Care Test for the Rapid Detection of Fecal Occult Blood: Instrument Validation Study
Source: J Med Internet Res. 2020 Aug 7;22(8):e20261. doi: 10.2196/20261 (PMC7472847; doi:10.2196/20261)
Supplement: Multimedia Appendix 2 [file jmir_v22i8e20261_app2.docx]

# **Supplementary Appendix**

### **Table S2. The primary interpretation result for the JustWipe^®^ and typical FOBT (O-tolidine-based test) in the central laboratory**

| Chart no. | Interpretation result | |
| --- | --- | --- |
|  | JustWipe | O-tolidine-based test at hospital |
| 1-1 | P | N |
| 1-2 | N | N |
| 1-3 | N | N |
| 1-4 | N | N |
| 1-5 | N | N |
| 1-6 | N | N |
| 1-7 | N | N |
| 1-8 | N | N |
| 1-9 | N | N |
| 1-10 | N | N |
| 1-11 | N | N |
| 1-12 | N | N |
| 1-13 | N | N |
| 1-14 | N | N |
| 1-15 | N | N |
| 1-16 | N | N |
| 1-17 | P | N |
| 1-18 | N | N |
| 1-19 | N | N |
| 1-20 | N | N |
| 1-21 | N | N |
| 1-22 | N | N |
| 1-23 | N | N |
| 1-24 | P | N |
| 1-25 | P | N |
| 1-26 | N | N |
| 1-27 | P | N |
| 1-28 | N | N |
| 1-29 | N | N |
| 1-30 | N | N |
| 1-31 | N | N |
| 1-32 | N | N |
| 1-33 | P | N |
| 1-34 | N | P |
| 1-35 | P | P |
| 1-36 | P | P |
| 1-37 | P | P |
| 1-38 | P | P |
| 1-39 | N | P |
| 1-40 | P | P |
| 1-41 | P | P |
| 1-42 | N | P |
| 1-43 | P | P |
| 1-44 | N | P |
| 1-45 | P | P |
| 1-46 | N | P |
| 1-47 | P | P |
| 1-48 | P | P |
| 1-49 | P | P |
| 1-50 | P | P |
| 1-51 | P | P |
| 1-52 | P | P |
| 1-53 | P | P |
| 1-54 | P | P |
| 1-55 | P | P |
| 1-56 | P | P |
| 1-57 | P | P |
| 1-58 | P | P |
| 1-59 | P | P |
| 1-60 | P | P |
| 1-61 | N | N |
| 1-62 | N | N |
| 1-63 | N | N |
| 1-64 | N | N |
| 1-65 | P | N |
| 1-66 | N | N |
| 1-67 | P | P |
| 1-68 | P | P |
| 1-69 | P | P |
| 1-70 | P | P |
