# Supplementary material for: Novel Toilet Paper–Based Point-Of-Care Test for the Rapid Detection of Fecal Occult Blood: Instrument Validation Study
Source: J Med Internet Res. 2020 Aug 7;22(8):e20261. doi: 10.2196/20261 (PMC7472847; doi:10.2196/20261)
Supplement: Multimedia Appendix 3 [file jmir_v22i8e20261_app3.docx]

# **Supplementary Appendix**

### **Table S3. The interpretation result of the individual patient on JustWipe^®^ and Hemoccult SENSA in the POCT setting**

| Object no. | Gender | Age | Interpretation result | |
| --- | --- | --- | --- | --- |
|  |  |  | JustWipe | Hemoccult  SENSA |
| 2-1 | M | 26 | P | P |
| 2-2 | F | 23 | N | N |
| 2-3 | M | 23 | N | N |
| 2-4 | F | 42 | N | N |
| 2-5 | F | 64 | N | N |
| 2-6 | M | 65 | P | P |
| 2-7 | F | 58 | N | N |
| 2-8 | F | 62 | N | N |
| 2-9 | M | 63 | P | P |
| 2-10 | F | 30 | N | N |
| 2-11 | F | 59 | N | N |
| 2-12 | F | 62 | P | P |
| 2-13 | F | 56 | P | P |
| 2-14 | M | 35 | N | N |
| 2-15 | M | 43 | N | N |
| 2-16 | F | 71 | N | P |
| 2-17 | M | 65 | P | P |
| 2-18 | M | 61 | N | N |
| 2-19 | F | 61 | P | P |
| 2-20 | F | 50 | P | P |
| 2-21 | M | 63 | N | P |
| 2-22 | F | 64 | N | N |
| 2-23 | M | 66 | N | N |
| 2-24 | F | 63 | N | N |
| 2-25 | F | 50 | N | N |
| 2-26 | M | 54 | N | N |
| 2-27 | F | 58 | N | N |
| 2-28 | M | 55 | N | N |
| 2-29 | M | 65 | P | P |
| 2-30 | F | 58 | N | N |
| 2-31 | F | 67 | N | N |
| 2-32 | M | 78 | N | N |
| 2-33 | M | 48 | P | P |
| 2-34 | F | 72 | P | P |
| 2-35 | M | 53 | N | N |
| 2-36 | M | 69 | N | N |
| 2-37 | F | 65 | P | N |
| 2-38 | F | 58 | N | N |
| 2-39 | F | 64 | P | N |
| 2-40 | F | 55 | N | N |
| 2-41 | F | 79 | N | N |
| 2-42 | F | 72 | P | P |
| 2-43 | F | 69 | P | P |
| 2-44 | M | 69 | N | N |
| 2-45 | M | 62 | N | N |
| 2-46 | M | 63 | N | N |
| 2-47 | F | 54 | N | N |
| 2-48 | F | 51 | P | P |
| 2-49 | M | 57 | N | P |
| 2-50 | F | 58 | N | N |
| 2-51 | M | 72 | N | N |
| 2-52 | M | 70 | P | P |
| 2-53 | M | 60 | N | P |
| 2-54 | F | 82 | N | N |
| 2-55 | M | 66 | N | N |
| 2-56 | M | 71 | P | P |
| 2-57 | F | 66 | N | N |
| 2-58 | F | 62 | N | N |
