# Supplementary material for: Novel Toilet Paper–Based Point-Of-Care Test for the Rapid Detection of Fecal Occult Blood: Instrument Validation Study
Source: J Med Internet Res. 2020 Aug 7;22(8):e20261. doi: 10.2196/20261 (PMC7472847; doi:10.2196/20261)
Supplement: Multimedia Appendix 4 [file jmir_v22i8e20261_app4.docx]

Table S5. **Association between individual usability indicators and result discordancy**. The table showed the contingency table for each question on recruited volunteers with discordance and concordance result.

|  |  | **Result compared betwwen Hemosensa and JustWipe^®^** | | |
| --- | --- | --- | --- | --- |
|  |  | Discordance | Concordance | p-value  (Fisher exact test) |
| Question 1 | Agree | 6 | 52 | 1 |
|  | Not Agree | 0 | 0 |  |
| Question 2 | Agree | 6 | 51 | 1 |
|  | Not Agree | 0 | 1 |  |
| Question 3 | Agree | 6 | 52 | 1 |
|  | Not Agree | 0 | 0 |  |
| Question 4 | Agree | 6 | 51 | 1 |
|  | Not Agree | 0 | 1 |  |
| Question 5 | Agree | 6 | 48 | 1 |
|  | Not Agree | 0 | 4 |  |
| Question 6 | Agree | 6 | 48 | 1 |
|  | Not Agree | 0 | 4 |  |
| Question 7 | Agree | 6 | 49 | 1 |
|  | Not Agree | 0 | 3 |  |
| Question 8 | Agree | 6 | 48 | 1 |
|  | Not Agree | 0 | 4 |  |
| Question 9 | Agree | 6 | 51 | 1 |
|  | Not Agree | 0 | 1 |  |
| Question 10 | Agree | 6 | 50 | 1 |
|  | Not Agree | 0 | 2 |  |
| Question 11 | Agree | 6 | 50 | 1 |
|  | Not Agree | 0 | 2 |  |
| Question 12 | Agree | 6 | 52 | 1 |
|  | Not Agree | 0 | 0 |  |
