# Supplementary material for: Novel Toilet Paper–Based Point-Of-Care Test for the Rapid Detection of Fecal Occult Blood: Instrument Validation Study
Source: J Med Internet Res. 2020 Aug 7;22(8):e20261. doi: 10.2196/20261 (PMC7472847; doi:10.2196/20261)
Supplement: Multimedia Appendix 6 [file jmir_v22i8e20261_app6.docx]

Table S4. **Demographic characteristics of recruited volunteer for POCT setting(n=58)**. The table contained the demographic characteristics of age, gender, education ,and occupation of the recruited volunteers.

| **Demographic characteristics** | | **Number(%)** | | | |
| --- | --- | --- | --- | --- | --- |
| **Age** | 20 - 29 | 3(5.2%) | | | |
|  | 30 - 39 | 2(3.4%) | | | |
|  | 40 - 49 | 3(5.2%) | | | |
|  | 50 - 59 | 15(25.9%) | | | |
|  | 60- 69 | 25(43.1%) | | | |
|  | > 70 | 10(17.2%) | | | |
| **Gender** | Female | 32(55.2%) | | | |
|  | Male | 26(44.9%) | | | |
| **Education** | Elementary school | 1(1.7%) | | | |
|  | Junio School | 8(13.8%) | | | |
|  | Senior School | 21(36.2%) | | | |
|  | University | 20(34.5%) | | | |
|  | Master/PhD degree | 8(13.8%) | | | |
| **Occupation** | Jobless | 17(29.3%) | | | |
|  | Retired | 1(1.7%) | | | |
|  | SOHO | 7(12%) | | | |
|  | Business | 3(5.2%) | | | |
|  | Accounting | 2(3.4%) | | | |
|  | Engineering | 3(5.2%) | | | |
|  | Student | 3(5.2%) | | | |
|  | Service industry | 3(5.2%) | | | |
|  | House keeping | 13(22.4%) | | | |
|  | Teacher | 2(3.4%) | | | |
|  | Sales representative | 1(1.7%) | | | |
|  | Labor | 2(3.4%) | | | |
